# Prognostic value of postoperative radiotherapy in patients with vulvar squamous carcinoma: findings based on the SEER database

**DOI:** 10.1186/s12905-023-02522-w

**Published:** 2023-07-08

**Authors:** Miaomiao Li, Jing Li, Zanhong Wang

**Affiliations:** Department of Obstetrics and Gynecology, Shanxi Bethune Hospital, Shanxi Academy of Medical Sciences， Tongji Shanxi Hospital, Third Hospital of Shanxi Medical University, No.99, Longcheng Street, Taiyuan, 030032 Shanxi China

**Keywords:** Vulvar squamous carcinoma, Surgery, SEER, Prognosis, Radiation therapy

## Abstract

**Introduction:**

The role of postoperative radiotherapy in treating squamous cell carcinoma of the vulva remains controversial. This study evaluated the effect of radiotherapy on the survival of patients with postoperative squamous cell carcinoma of the vulva.

**Methods:**

Clinical and prognostic information on patients diagnosed with vulvar squamous cell carcinoma from 2010 to 2015 was collected from the Surveillance, Epidemiology, and Prognosis (SEER) database. A propensity score matching (PSM) approach was used to balance the differences in clinicopathological characteristics between groups. The impact of postoperative radiotherapy on overall survival (OS) and disease-specific survival (DSS) was assessed.

**Results:**

The study included 3571 patients with squamous cell carcinoma of the vulva, of whom 732 (21.1%) received postoperative radiotherapy. After propensity score matching, multivariate analysis showed that age, race, N stage, and tumor size were independent influences on overall survival and disease-specific survival of patients. Postoperative radiotherapy did not improve patients’ overall survival or disease-specific survival. Further subgroup survival analysis showed that in patients with AJCC stage III, N1 stage, lymph node metastasis, and large tumor diameter (> 3.5 cm), postoperative radiotherapy resulted in a significant improvement in overall patient survival.

**Conclusion:**

Postoperative radiotherapy is not indicated for all patients with postoperative vulvar cancer and has improved survival outcomes only for patients with AJCC stage III, N1, lymph node metastases and large tumor diameter (> 3.5 cm).

**Supplementary Information:**

The online version contains supplementary material available at 10.1186/s12905-023-02522-w.

## Introduction

Vulvar cancer is a rare gynecologic malignancy, accounting for 5% of gynecologic malignancies, and it is most common in older women [1]. Over the past decade, the overall incidence of vulvar cancer has increased by an average of 4.6% every five years [2]. According to reports, there were 6120 new vulvar cancer cases in the United States in 2020 and 1350 deaths [3]. The most common pathological type of vulvar cancer is vulvar squamous cell carcinoma, followed by melanoma. Vulvar bleeding, pain, and sexual dysfunction are the main clinical symptoms of the disease, which seriously affect the quality of life and physical and mental health of patients.

Surgery is the primary method of treatment for vulvar cancer. Compared to radical vulvectomy, partial radical vulvectomy has become the procedure of choice in recent years due to fewer postoperative complications [4–6]. The main reasons currently affecting the prognosis of patients with vulvar cancer are postoperative complications and a high rate of local recurrence. Although postoperative radiotherapy is thought to enhance the control of postoperative tumors, the role of postoperative radiotherapy is not fully understood due to the low incidence of vulvar cancer and limited clinical studies in large samples.

This study explored the impact of postoperative radiotherapy on the prognosis of patients with vulvar squamous cell carcinoma using data from the SEER database. To minimize selection bias in the included sample, propensity score matching was performed to balance the distribution of baseline clinicopathological variables between the two cohort populations.

## Materials and methods

Patient data for this study were obtained from the National Cancer Institute’s SEER database between 2010 and 2015. The SEER database is a sizeable cancer-related database in the United States [7, 8], containing information on the incidence, treatment, and prognosis of cancer patients collected by multiple institutions since 1973. We used SEER*Stat software (version 8.3.9) to extract eligible cases from the database.

For patients with pathologically confirmed vulvar cancer from 2010 to 2015, the primary vulvar site cases were obtained using the “primary site” variable. The histological subtype of vulvar cancer was determined using the variable “ICD-0-3 Hist/Behav, malignant”. We extracted demographic variables, including age at diagnosis, race, primary site, histological type, tumor grade, tumor size, surgery, radiotherapy, chemotherapy, lymph node status, marital status, cause of death, survival (from diagnosis to end or last follow-up), TNM staging, and AJCC staging (7th edition).

Since the distribution of patients in the SEER database is not random, selection bias of baseline characteristics may affect the final results. To reduce the effects of data bias and confounding variables, propensity score matching (PSM) was used to adjust for potential baseline confounders [9]. Scores were calculated based on the nearest neighbor 1:1 matching algorithm, while logistic regression was used to construct the model. The following baseline covariates were considered: patient age, race, pathological grade, tumor grade, tumor size, T-stage, N-stage, M-stage, whether lymph nodes were metastatic, and marital status, with a set caliper of 0.5, and patients who received postoperative radiotherapy were matched to other patients.

Inclusion criteria for this study: (1) patients without distant metastases at diagnosis; (2) positive histopathological confirmation; (3) undergoing surgical treatment. Exclusion criteria: (1) incomplete patient data, such as age at diagnosis, radiotherapy records, survival status and duration; (2) not treated with surgery; (3) non-squamous cell carcinoma pathological types; (4) patients with distant metastases from vulvar cancer.

**Fig. 1:**
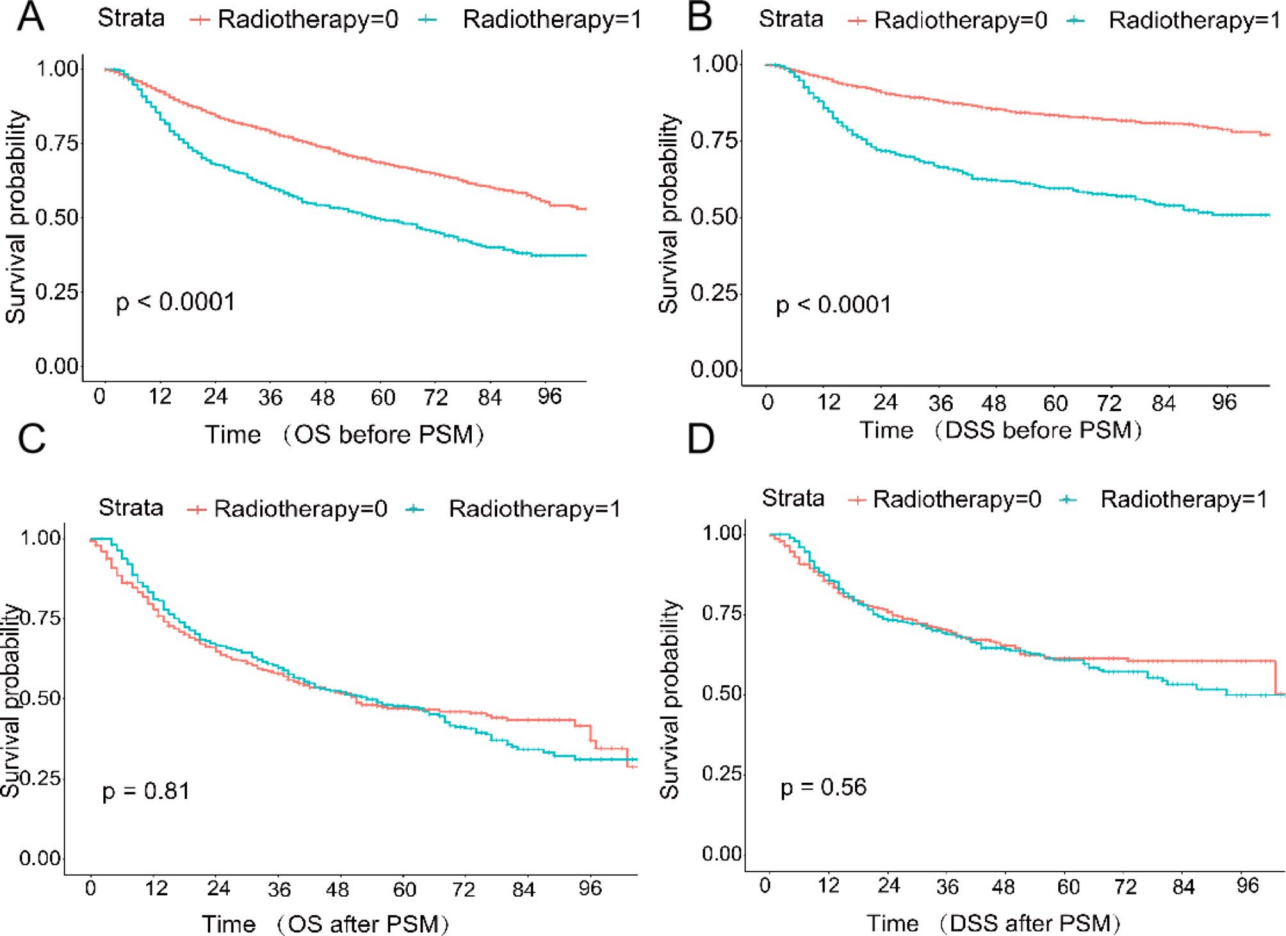
Comparison of overall survival OS **(A)** and disease-specific survival DSS **(B)** between patients in the post-operative radiation therapy and no post-operative radiation therapy groups before PSM;Comparison of overall survival OS **(C)** and disease-specific survival DSS **(D)** between patients in the post-operative radiation therapy (PORT) and no post-operative radiation therapy groups after PSM.

Based on the above criteria, 3571 cases were finally included in this study (Fig. 1 and Additional File Fig S1). Based on the definition provided by SEER, distant metastases are defined as metastases of vulvar cancer lesions to the bladder or rectum, the proximal 2/3 of the urethra, the pelvis, and/or distant lymph nodes. Because SEER provides limited information on specific treatment, surgery includes all types of surgery and radiation therapy includes all types of radiation therapy.

The tumors were classified into four different size groups by ranking the lesion sizes of the cases included in this study, using the interquartile spacing as the cut-off value, in which > 75% of the tumors (> 3.5 cm in diameter) were defined as large tumors, which is closer to the locally advanced tumors (> 4 cm in diameter) defined in the 2022 NCCN vulvar cancer guidelines, and also more in line with the actual situation of this study.

The primary and secondary endpoints were overall survival (OS) and disease-specific survival (DSS). Categorical variables were analyzed using the chi-square test. The Kaplan-Meier method was used to produce survival curves and the log-rank test to analyze the differences between the survival outcomes of the two curves. Univariate and multivariate Cox scale risk models were used to identify risk factors affecting OS and DSS. A Cox proportional risk model was used in the subgroup analysis to determine the population of patients who might benefit from radiotherapy. It is considered statistically significant when the bilateral p-value is less than 0.05. The above statistical analysis was calculated using SPSS software (version 24.0) and R software (version 4.0.2).

## Results

This study included 3571 patients who underwent surgery and were diagnosed with vulvar squamous cell carcinoma between 2010 and 2015, of whom 732 (20.4%) received radiotherapy. Table 1 shows the demographic and clinicopathological characteristics between the group receiving postoperative radiotherapy and the group not receiving postoperative radiotherapy. Prior to propensity score matching (PSM), significant differences existed between the two groups in terms of race, tumor cell grading, disease stage, tumor size, chemotherapy status, lymph node metastasis, and lymph node surgical distribution. After balancing the baseline characteristics of the two PSM groups, there were 385 patients in each group. All characteristics were balanced between the cohorts using matched propensity scores (Table 1).

**Table 1:** Patient information based on their baseline features before and after 1:1 PSM in the radiotherapy and non-radiotherapy groups

Characteristic	Before PSM					After PSM			
	Total no.	PROT(-) No(%)	PROT(+) No(%)	P-value		Total no.	PROT(-) No(%)	PROT(+) No(%)	P-value
**All patients**	3571	2839	732			770	385	385	
**Age(years)**									
≤ 49	558	459(12.9)	99(2.8)	0.056		71	35(4.5)	36(4.7)	0.451
50–69	1532	1192(33.4)	340(9.5)			298	141(18.3)	157(20.4)	
≥ 70	1481	1188(33.3)	293(8.2)			401	209(27.1)	192(24.9)	
**Race**									
White	3161	2528(70.8)	633(17.7)	0.054		595	262(34.0)	333(43.2)	< 0.001
Black	299	232(6.5)	67(6.5)			102	71(9.2)	31(4.0)	
Other	111	79(2.2)	32(0.9)			73	52(6.8)	21(2.7)	
**Marital statues**									
Married	1359	1071(30.1)	288(8.1)	0.421		264	120(15.6)	144(18.7)	0.068
Other	2212	1768(49.5)	444(12.4)			506	265(34.3)	241(31.3)	
**Grade**									
I	1091	969(27.1)	122(3.4)	< 0.001		137	73(9.5)	64(8.3)	0.871
II	1378	1024(28.7)	354(9.9)			359	179(23.2)	180(23.4)	
III	480	287(8.1)	193(5.4)			210	103(13.4)	107(13.9)	
IV	24	17(0.5)	7(0.2)			8	3(0.4)	5(0.6)	
Unknown	598	542(15.2)	56(1.6)			73	27(3.5)	29(3.8)	
**AJCC stage**									
I	2787	2568(71.9)	219(6.1)	< 0.001		346	169(21.9)	177(23.0)	0.832
II	156	86(2.4)	70(2.0)			72	34(4.4)	38(4.9)	
III	560	161(4.5)	399(11.2)			307	158(20.5)	149(19.4)	
IV	68	24(0.7)	44(1.2)			58	24(3.1)	21(2.7)	
**T**									
T1	3272	2711(75.9)	561(15.7)	< 0.001		621	310(40.3)	311(40.4)	0.389
T2	249	140(3.9)	109(3.1)			118	56(7.3)	62(8.1)	
T3	50	19(0.5)	31(0.9)			31	19(2.5)	12(1.6)	
**N**									
N0	2975	2666(74.7)	309(8.7)	< 0.001		435	215(27.9)	221(28.7)	0.881
N1	318	99(2.8)	219(6.1)			188	98(12.7)	90(11.7)	
N2	255	67(1.9)	188(5.3)			130	65(8.4)	65(8.4)	
N3	23	7(0.2)	16(0.4)			16	7(0.9)	9(1.2)	
**Tumor size(cm)**									
≤ 0.3(25%)	909	833(23.3)	76(2.1)	< 0.001		68	27(3.5)	41(5.3)	0.255
0.3–1.7(50%)	926	819(22.9)	107(3.0)			135	64(8.3)	71(9.2)	
1.7–3.5(75)	830	637(17.8)	193(5.4)			187	98(12.7)	89(11.6)	
＞3.5	906	550(15.4)	356(10.0)			196	196(25.5)	184(23.9)	
**Chemotherapy**									
Yes	3206	2807(78.6)	399(11.2)	< 0.001		692	353(45.8)	339(44.0)	0.094
No	365	333(9.3)	32(0.9)			78	32(4.2)	46(6.0)	
**LN metastasis**									
Yes	3900	487(10.5)	271(5.8)	< 0.001		437	216(28.1)	221(28.7)	0.716
No	758	344(7.4)	3556(76.3)			333	169(21.9)	164(21.3)	
**Surgery to LN**									
No	1664	1448(40.5)	216(6.0)	< 0.001		257	129(16.8)	128(16.6)	0.591
lymph node biopsy	339	268(7.5)	71(2.0)			68	30(3.9)	38(4.9)	
lymphadenectomy	1568	1123(31.4)	445(12.5)			445	226(29.4)	219(38.4)	

PORT: postoperative radiotherapy; PSM: propensity score matching; LN: Lymph nodes

Before PSM, the 5-year OS and DSS were significantly higher in the no-radiotherapy group (OS: 68.5%; DSS: 88.5%) than in the radiotherapy group (OS: 48.6%; DSS: 62.0%, both P < 0. 001); there were multiple significant crossover points in the K-M survival analysis curves between the radiotherapy and no-radiotherapy groups after PSM, suggesting that the model was a non-equivalent regression model, and by log - rank test, there was no significant difference in OS and DSS between the radiotherapy and no-radiation groups (P = 0.81, P = 0.56) (Fig. 1). Multivariate analysis showed (Tables 2 and 3) that postoperative radiotherapy had no significant effect on OS (HR 1.138, 95% CI 0.940–1.377, P value 0.186) and DSS (HR 1.006, 95% CI 0.797–1.270, P value 0.959) of patients. Age (HR 2.027, 95% CI 1.698–2.420, p < 0.001; HR 1.937, 95% CI 1.555–2.412,p < 0.001), race (HR 0.792, 95% CI 0.666–0.942, p = 0.008; HR 0.713, 95% CI 0.564–0.901, p = 0.005), AJCC staging (HR 1.199, 95% CI 1.014–1.419, p = 0.034; HR 1.445, 95% CI 1. 191-1.752, p < 0.001), N staging (HR 1.417, 95% CI 1.140–1.760, p = 0.002; HR 1.579 95% CI 1. 191-1.937, p = 0.001) and tumor size (HR 1.240, 95% CI 1.113–1.381, p < 0.001; HR 1.192, 95% CI 1.041–1.365, p = 0. 011) were independent factors affecting patients’ OS and DSS.

**Table 2:** Univariate and multivariate Cox regression analyses of different variables considered for OS for patients with carcinoma of vulva

Characteristic	Univariate			Multivariate	
	HR(95%CI)	P-value		HR(95%CI)	P-value
**Age(years)**					
≤ 49	1			1	
50–69	0.280(0.183–0.429)	＜0.001		0.335(0.215–0.520)	＜0.001
≥ 70	0.369(0.297–0.458)	＜0.001		0.439(0.349–0.551)	＜0.001
**Race**					
White	1			1	
Black	1.788(1.225–2.611)	0.003		1.614(1.099–2.370)	0.015
Other	1.059(0.665–1.685)	0.811		1.442(0.893–2.329)	0.135
**Marital statues**	0.712(0.581–0.874)	0.001		0.889(0.714–1.107)	0.292
**Grade**					
I	1			1	
II	1.090(0.689–1.725)	0.711		1.225(0.753–1.992)	0.413
III	1.468(0.969–2.222)	0.071		1.392(0.893–2.171)	0.144
IV	1.625(1.060–2.492)	0.026		1.350(0.850–2.144)	0.204
Unknown	1.294(0.450–3.721)	0.632		1.540(0.520–4.560)	0.436
**AJCC stage**					
I	1			1	
II	0.403(0.275–0.590)	＜0.001		2.935(0.331–26.025)	0.333
III	0.637(0.405–1.003)	0.052		3.600(0.393–32.983)	0.257
IV	0.856(0.590–1.242)	0.413		2.079(0.230-18.829)	0.515
**T**					
T1	1			1	
T2	0.539(0.353–0.823)	0.004		0.233(0.028–1.912)	0.175
T3	0.745(0.468–1.185)	0.214		0.238(0.029–1.982)	0.184
**N**					
NO	1			1	
N1	0.418(0.228–0.767)	0.005		0.113(0.014–0.901)	0.041
N2	0.654(0.352–1.214)	0.178		0.308(0.037–2.545)	0.274
N3	1.112(0.597–2.074)	0.738		0.458(0.056–3.717)	0.465
**Tumor size(cm)**					
≤ 0.3(25%)	1			1	
0.3–1.7(50%)	0.585(0.407–0.840)	0.004		0.722(0.490–1.064)	0.101
1.7–3.5(75%)	0.443(0.331–0.593)	＜0.001		0.569(0.418–0.776)	＜0.001
＞3.5	0.574(0.453–0.728)	＜0.001		0.664(0.517–0.851)	＜0.001
**Radiotherapy**	1.077(0.893–1.298)	0.439		1.138(0.940–1.377)	0.186
**Chemotherapy**	1.304(0.935–1.819)	0.118		1.217(0.838–1.768)	0.302
**LN metastasis**	0.518(0.429–0.625)	＜0.001		1.136(0.068–19.093)	0.931
**Surgery to LN**					
NO	1			1	
lymph node biopsy	0.854(0.695–1.048)	0.131		1.506(1.156–1.961)	0.002
lymphadenectomy	0.951(0.674–1.342)	0.775		1.156(0.811–1.649)	0.422

CI: confidence interval; OS: overall survival; HR: hazard ratio; PORT: postoperative radiotherapy

**Table 3:** Univariate and multivariate Cox regression analyses of different variables considered for DSS for patients with carcinoma of vulva

Characteristic	Univariate			Multivariate	
	HR(95%CI)	P-value		HR(95%CI)	P-value
**Age(years)**					
≤ 49	1			1	
50–69	0.277(0.161–0.478)	＜0.001		0.338(0.192–0.595)	＜0.001
≥ 70	0.402(0.309–0.524)	＜0.001		0.498(0.376–0.660)	＜0.001
**Race**					
White	1			1	
Black	2.068(1.265–3.382)	0.004		1.816(1.104–2.989)	0.019
Other	0.792(0.415–1.5112)	0.479		1.188(0.610–2.312)	0.613
**Marital statues**	0.734(0.570–0.945)	0.016		0.882(0.671–1.160)	0.371
**Grade**					
I	1			1	
II	1.003(0.562–1.791)	0.992		1.343(0.721–2.501)	0.353
III	1.459(0.869–2.451)	0.153		1.547(0.882–2.713)	0.128
IV	1.775(1.043–3.018)	0.034		1.566(0.877–2.796)	0.129
Unknown	0.985(0.226–4.286)	0.984		1.265(0.280–5.708)	0.761
**AJCC stage**					
I	1			1	
II	0.208(0.137–0.316)	＜0.001		1.318(0.146–11.933)	0.806
III	0.350(0.206–0.597)	＜0.001		1.518(0.160-14.404)	0.716
IV	0.640(0.435–0.942)	0.024		1.880(0.202–17.539)	0.579
**T**					
T1	1			1	
T2	0.352(0.227–0.547)	＜0.001		0.266(0.032–2.212)	0.221
T3	0.517(0.314–0.851)	0.009		0.303(0.036–2.567)	0.274
**N**					
NO	1			1	
N1	0.222(0.119–0.412)	＜0.001		0.098(0.012–0.799)	0.031
N2	0.448(0.238–0.841)	0.012		0.250(0.029–2.137)	0.205
N3	0.851(0.453–1.601)	0.617		0.407(0.049–3.398)	0.407
**Tumor size(cm)**					
≤ 0.3(25%)	1			1	
0.3–1.7(50%)	0.573(0.364–0.902)	0.016		0.814(0.499–1.329)	0.411
1.7–3.5(75%)	0.419(0.290–0.605)	＜0.001		0.622(0.420–0.922)	0.018
＞3.5	0.557(0.414–0.749)	＜0.001		0.693(0.506–0.948)	0.022
**Radiotherapy**	1.073(0.846–1.359)	0.562		1.006(0.797–1.270)	0.959
**Chemotherapy**	0.936(0.651–1.346)	0.721		0.928(0.612–1.407)	0.725
**LN metastasis**	0.375(0.296–0.477)	＜0.001		1.705(0.099–29.313)	0.713
**Surgery to LN**					
NO	1			1	
lymph node biopsy	0.659(0.504–0.861)	0.002		1.352(0.959–1.908)	0.086
lymphadenectomy	0.900(0.593–1.368)	0.623		1.159(0.753–1.785)	0.502

CI: confidence interval; HR: hazard ratio; DSS: disease specific survival; PORT: postoperative radiotherapy

**Fig. 2:**
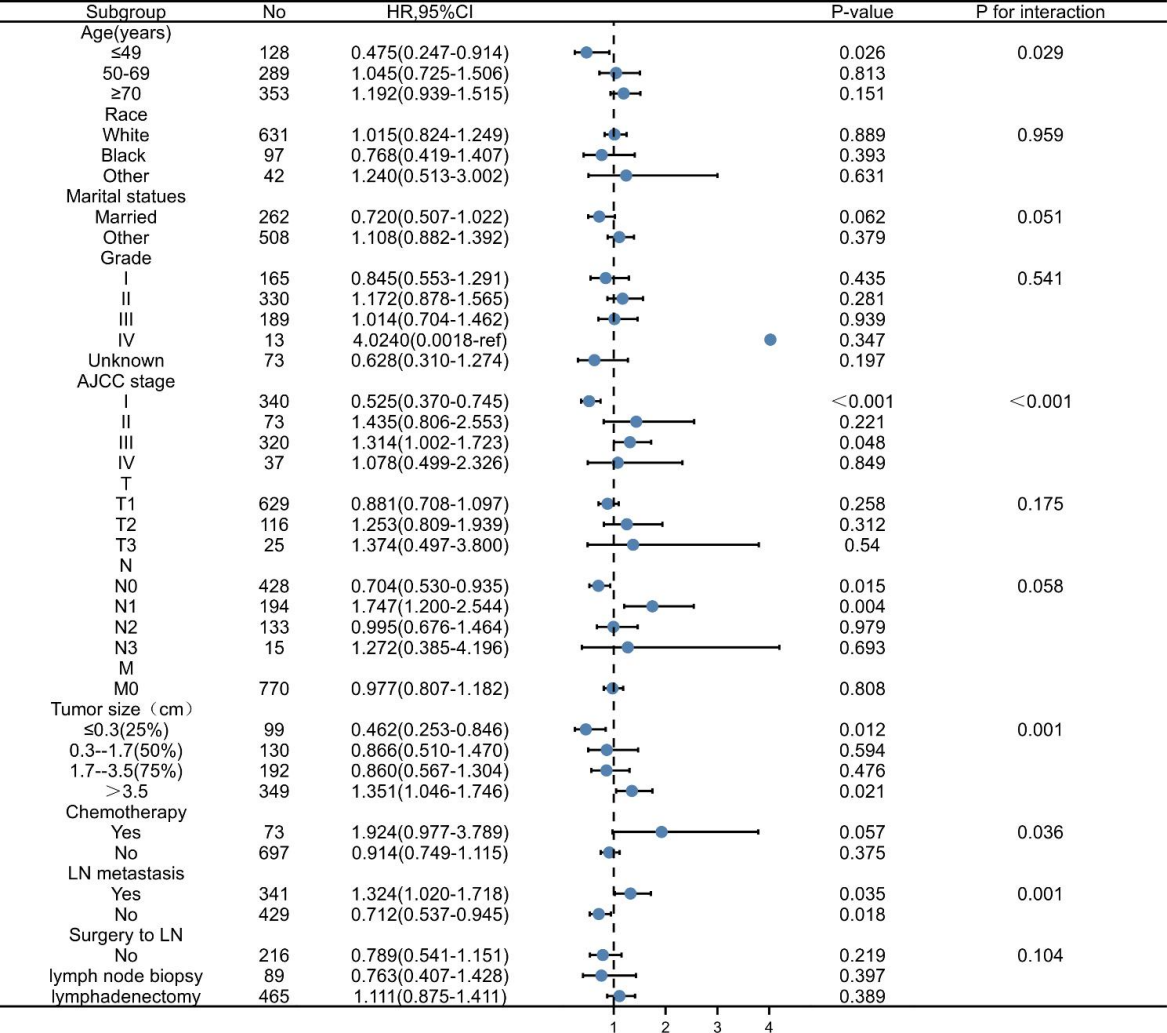
The forest plot of HRs comparing patients with postoperative vulvar cancer between the PORT group and no-PORT group according to different variables

**Fig. 3:**
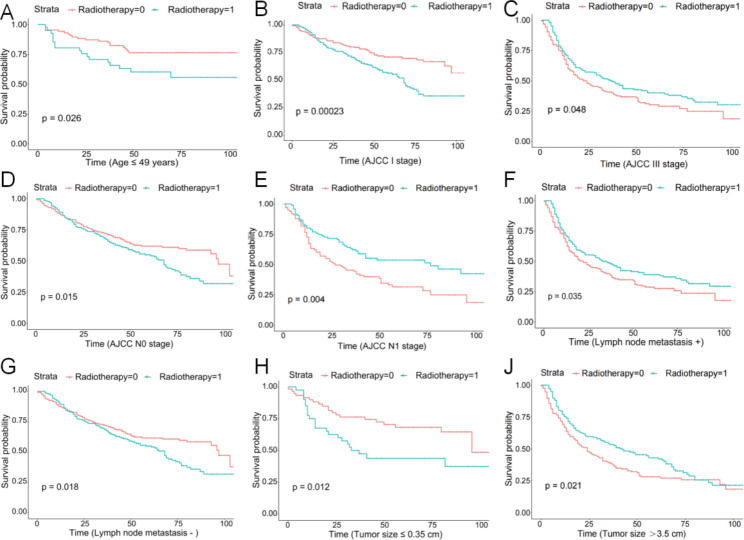
Summary of survival curves for subgroup analysis. **A:** Comparison of survival outcomes in patients aged less than 49 years who received postoperative radiotherapy with or without; **B:** Comparison of survival outcomes in AJCC stage I patients treated with and without postoperative radiotherapy; **C:** Comparison of survival outcomes in AJCC stage III patients treated with and without postoperative radiotherapy; **D:** Comparison of survival outcomes of AJCC stage N0 patients treated with or without postoperative radiotherapy; **E:** Comparison of survival outcomes in AJCC stage N1 patients treated with or without postoperative radiotherapy; **F:** Comparison of survival outcomes in patients with lymph node metastases treated with and without postoperative radiotherapy; **G:** Comparison of survival outcomes in patients with non-metastatic lymph nodes treated with and without postoperative radiotherapy; **H:** Comparison of survival outcomes in patients with small tumor lesions treated with and without postoperative radiotherapy; **J:** Comparison of survival outcomes in patients with large tumor lesions treated with and without postoperative radiotherapy

Subgroup analysis showed a significant benefit of postoperative radiotherapy in improving OS in patients with AJCC grade III and N1 (P = 0.048, P = 0.004). For patients with lymph node metastasis, postoperative radiotherapy significantly improved OS (P = 0.035). For patients with large tumor size (> 3.5 cm in diameter), postoperative radiotherapy had better OS (P = 0.021) (Figs. 2 and 3).

## Discussion

Owing to the low incidence of vulvar squamous cell carcinoma, it is challenging to study the prognostic impact of postoperative radiotherapy on patients with vulvar squamous cell carcinoma using large randomized controlled trials [10]. In recent years, oncologic radiotherapy techniques have developed rapidly [11, 12]. Their role in treating patients with vulvar cancer has received increasing attention [13]; however, the effect of postoperative radiotherapy on the survival of patients with squamous cell carcinoma of the vulva is unclear.

Due to differences in tumor biology, several previous single-center studies have reported the role of radiotherapy in treating patients with vulvar cancer [14]. Ignatov T et al. recruited 257 patients with vulvar squamous cell carcinoma and divided the enrolled patients into lymph node metastasis positive and negative groups and found that adjuvant radiotherapy did not improve the prognosis of lymph node negative patients [15]. Meanwhile, Macit Arvas et al. studied 107 patients with postoperative vulvar cancer with long-term follow-up. They found that postoperative radiotherapy only improved the 5-year overall survival of patients with positive surgical margins [16].

To obtain data closely related to current clinical practice, clinical information on patients with vulvar cancer from the SEER database from 2010 to 2015 was collected for this study, and PSM was used to balance the clinical characteristics of the two samples, which could more closely resemble a randomized controlled study and make the results more reliable. According to the inclusion criteria, 3571 patients with squamous cell carcinoma of the vulva were included in this study. The effect of postoperative radiotherapy on survival could be directly analyzed by 1:1 PSM matching according to whether they received postoperative radiotherapy or not. The results showed that postoperative radiotherapy failed to improve OS and DSS in patients with vulvar squamous carcinoma.

Regarding prognostic factors of vulvar cancer, VULCAN retrospectively analyzed the clinicopathological characteristics and prognosis of 1727 patients with vulvar cancer. Multivariate analysis showed that tumor stage, tumor size and ethnicity profile were independent prognostic factors affecting patients’ OS. In addition, the French Society of Radiation Oncology specified that the main factors affecting the postoperative prognosis of patients with vulvar cancer include lymph node involvement, tumor stage, and patient age [17]. Our cohort underwent multivariate COX regression analysis after PSM analysis, and tumor size was also a factor influencing patient prognosis; however, postoperative radiotherapy in patients with squamous vulvar cancer was not an independent influencing factor.

To further clarify the subgroup population benefiting from postoperative radiotherapy for vulvar cancer and to guide personalized clinical treatment, we conducted a subgroup survival analysis, which showed that among patients receiving radiotherapy, OS was significantly higher in patients with AJCC stage III, N1 stage, lymph node metastasis, and large tumor diameter. A recent study analyzing factors related to overall survival and disease recurrence in patients with vulvar cancer pointed out that the size of the primary lesion is an important reference for overall survival [18]. Tumor diameter is closely related to lymph node metastasis. When the lesion diameter is less than 2 cm, the lymph node metastasis rate is about 23%, while when the lesion diameter is greater than 2 cm, the lymph node metastasis rate is as high as 47% [19]. Adjuvant radiotherapy has been shown to significantly prolong the overall survival of patients with advanced disease [17, 20]. In a retrospective analysis of 54 cases of vulvar cancer by S C Han 1 et al., adjuvant radiotherapy was found to significantly improve disease-specific survival (P = 0.03) and overall survival (P = 0.04) in patients with locally advanced vulvar cancer [21]. In this study, survival analysis of a PSM-matched cohort showed a significant improvement in overall survival after postoperative radiotherapy in patients with advanced (AJCC stage III) vulvar cancer, which may be related to the better control of disease recurrence with adjuvant radiotherapy.

It has been confirmed that lymph node status, including whether the lymph nodes are metastatic and the number of positive lymph nodes, affects the recurrence of vulvar cancer and the prognosis of patients [22, 23]. Meanwhile, the International Federation of Obstetrics and Gynecology guidelines recommend adjuvant radiotherapy as a necessary treatment for lymph node-positive vulvar cancer patients. For patients with lymph node-positive vulvar cancer, adjuvant radiotherapy was associated with a lower risk of local recurrence (25.5% vs. 15.8%) [24]. A recent AGO-CaRE 1 study found that adjuvant radiotherapy significantly improved progression-free survival in lymph node-positive vulvar cancer [10].

Our study has some limitations: first, it is a retrospective study with some selection bias, and PSM minimized the influence of confounding factors on the results. Secondly, the SEER database does not contain indicators of specific radiotherapy methods, radiotherapy sites, and radiotherapy regimens, which also affect patient prognosis.

In conclusion, an analysis of vulvar cancer data from the SEER database showed that postoperative radiotherapy failed to improve overall and disease-specific survival in all patients with vulvar squamous cell carcinoma. Postoperative radiotherapy improved survival outcomes only in patients with AJCC stage III, N1, large tumor diameter, and lymph node metastases.

## Electronic supplementary material

Below is the link to the electronic supplementary material.

Additional File Fig S1: The inclusion criteria flowchart of recruited patients in SEER database

## Data Availability

Data from the SEER program is available for public. The data supporting the conclusions of this article are available in the Surveillance Epidemiology, and End Results (SEER) database (https://seer.cancer.gov/).
