# Supplementary figures and images for: Prognostic value of postoperative radiotherapy in patients with vulvar squamous carcinoma: findings based on the SEER database
Source: BMC Womens Health. 2023 Jul 8;23:361. doi: 10.1186/s12905-023-02522-w (PMC10329365; doi:10.1186/s12905-023-02522-w)

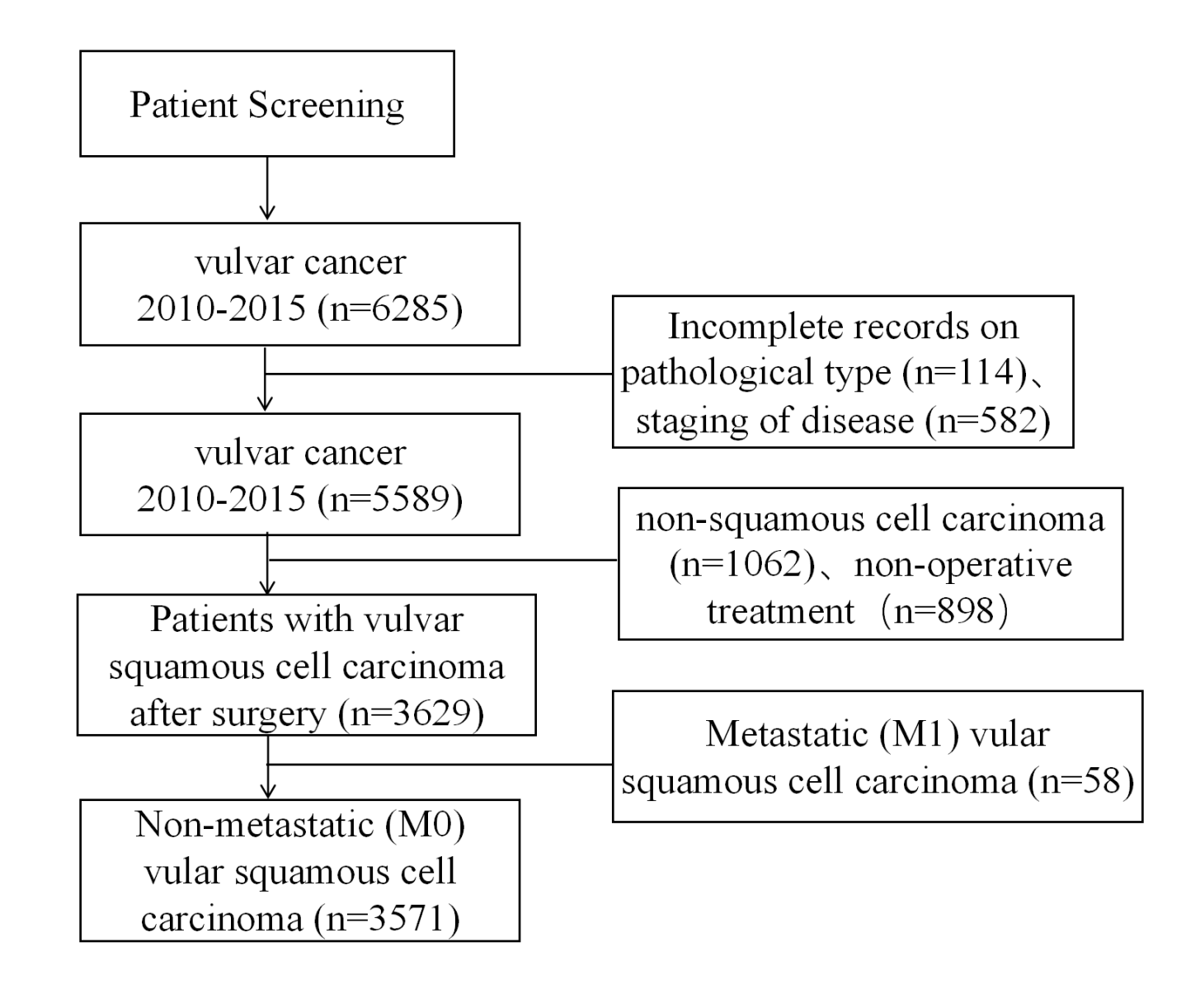
Supplementary Fig S1: The inclusion criteria flowchart of recruited patients in SEER database.

Supplement: Supplementary file 1 — Additional File Fig S1: The inclusion criteria flowchart of recruited patients in SEER database [file 12905_2023_2522_MOESM1_ESM.docx]
